# Correction: Dynamic Maternal Gradients Control Timing and Shift-Rates for *Drosophila* Gap Gene Expression

**DOI:** 10.1371/journal.pcbi.1005918

**Published:** 2017-12-22

**Authors:** The PLOS Computational Biology Staff

There is a typographical error in equation 3 in the main manuscript. The text should read as follows:

$$u_{i}^{a}(t)=\sum_{b\in G}W^{ba}g_{i}^{b}(t)+\sum_{m\in M}E^{ma}g_{i}^{m}(t)+h^{a}$$

Moreover, we accidentally misreported the root mean square (RMS) score of the non-autonomous diffusion-less gap gene circuit we analyze in this paper. As a consequence, the last paragraph of the section “Model Fitting and Selection” on page 8 of our article should read (revised text highlighted in blue):

The residual error of our best-fitting diffusion-less circuit (RMS = 14.53) lies at the upper end of the range of residual errors for fully-non-autonomous circuits with diffusion, which range from RMS scores of 10.43 to 13.32 [26]. The small increase in RMS score, and the fact that all gap domains are reproduced with accurate timing and position in our circuit, lends further support to the notion that diffusion is not essential for gap gene patterning. Moreover, our previous work also shows that circuits which were fit without weighting the data show somewhat lower RMS scores of 8.71 to 10.11 despite exhibiting more patterning defects at late stages [26]. The RMS score of the static-Bcd model (fit without weights) is slightly higher, at 10.76 [22]. In this sense, the effect of switching off diffusion is similarly negligible in static-Bcd and fully non-autonomous models. A visual inspection of circuits indicates that non-autonomous circuits are able to produce smoother domain boundaries than autonomous ones (compare our Fig 3B with Fig 2H,J in Manu *et al*. [22]).
