# Supplementary material for: Dynamic Maternal Gradients Control Timing and Shift-Rates for Drosophila Gap Gene Expression
Source: PLoS Comput Biol. 2017 Feb 3;13(2):e1005285. doi: 10.1371/journal.pcbi.1005285 (PMC5291410; doi:10.1371/journal.pcbi.1005285)
Supplement: S1 Table — Model equations are shown in the Models and Methods section. (PDF) [file pcbi.1005285.s001.pdf]

Promoter Strengths		R^{hb}	R^{Kr}	R^{gt}	R^{kni}
		10.0001	16.9328	15.9656	13.0138
Interconnectivity Matrix (W)		**hb**	**kr**	**gt**	**kni**
	hb	0.0102	0.0000	0.0246	-0.2625
	kr	-0.0040	0.0113	-0.2723	-0.0106
	gt	-0.0060	-0.3882	0.0047	0.0162
	kni	-0.1821	0.0000	-0.0642	0.0055
External Input Strengths (E)		**bcd**	**cad**	**hkb**	**tll**
	hb	0.0790	0.0016	0.1702	-0.8470
	kr	0.0691	0.0244	-3.1254	0.0000
	gt	0.0912	0.0258	-0.0947	0.0000
	kni	0.0000	0.0276	-0.7241	0.0000
Promoter Thresholds		h^{hb}	h^{Kr}	h^{gt}	h^{kni}
		-2.5000	-2.5000	-2.5000	-2.5000
Protein Half Lives		**Hb**	**Kr**	**Gt**	**Kni**
		15.4725	9.6659	9.6622	19.9993
Diffusion Parameters		D^{hb}	D^{Kr}	D^{gt}	D^{kni}
		0.0000	0.0000	0.0000	0.0000
