# Supplementary figures and images for: Dynamic Maternal Gradients Control Timing and Shift-Rates for Drosophila Gap Gene Expression
Source: PLoS Comput Biol. 2017 Feb 3;13(2):e1005285. doi: 10.1371/journal.pcbi.1005285 (PMC5291410; doi:10.1371/journal.pcbi.1005285)

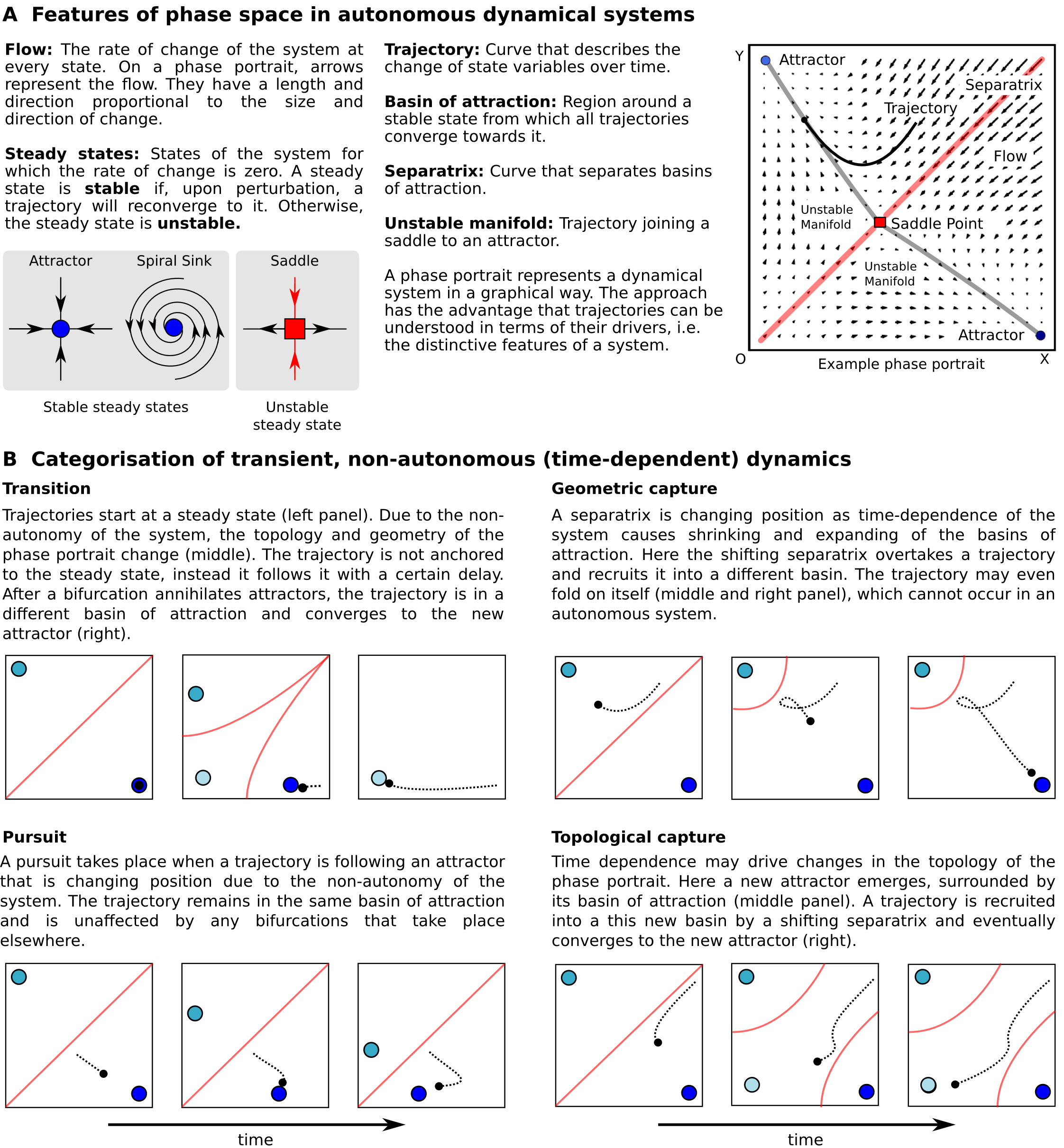

Supplement: S1 Fig — (A) Features of phase space in autonomous dynamical systems. (B) Categorisation of transient, non-autonomous dynamics. (PNG) [file pcbi.1005285.s002.png]

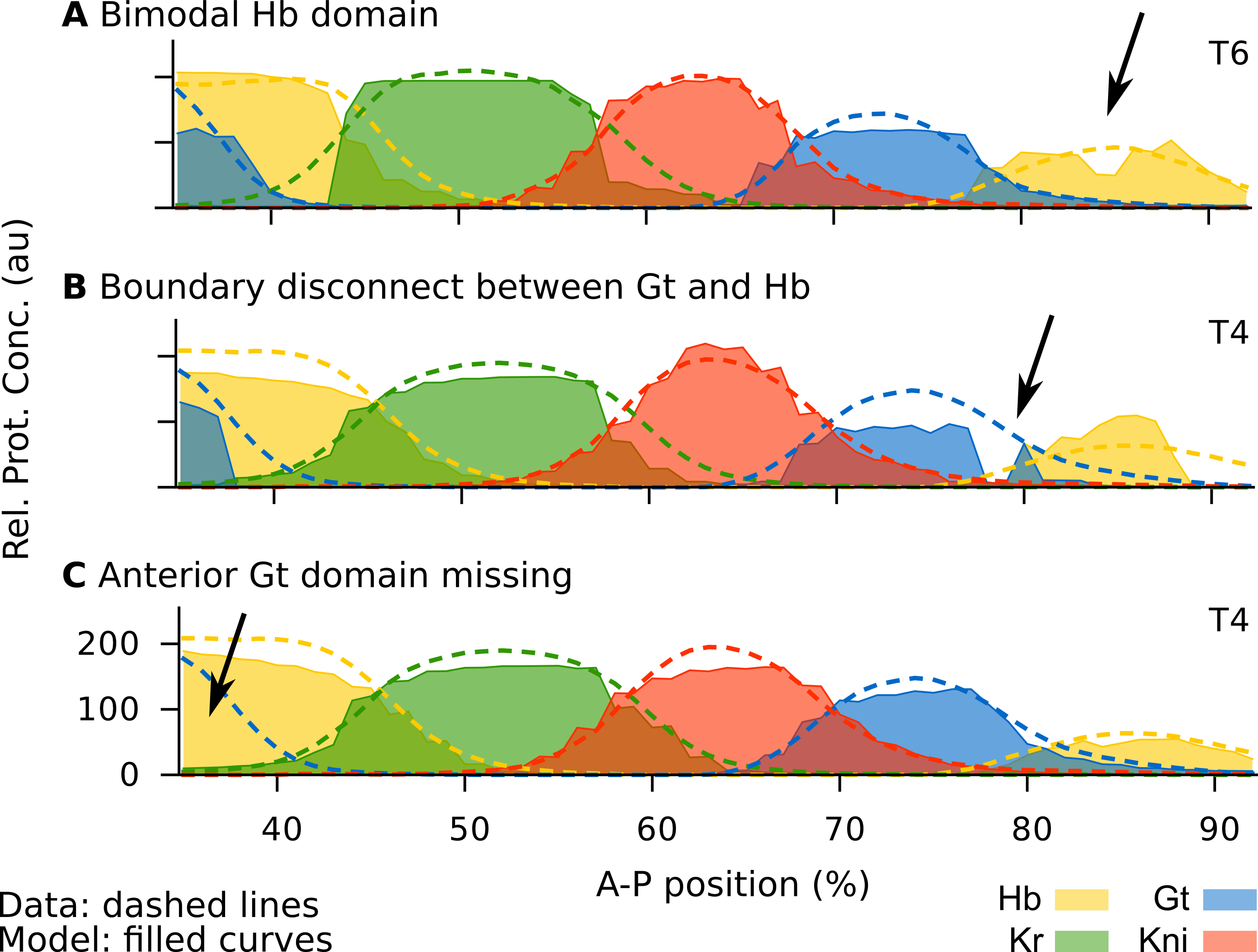

Supplement: S2 Fig — Commonly observed defects in fully autonomous D. melanogaster gap gene circuits fitted to data without diffusion. Circuits showing any of these gross patterning defects were excluded from further analysis, even if their RMS score was low. Arrows indicate patterning defects as named in the panel headings (A–C). Horizontal axes represent %A–P position (where 0% is the anterior pole). Vertical axes show relative protein expression levels (Rel. Prot. Expr.) in arbitrary units (au). T4/6 indicate time classes C14-T4 and T6, respectively. (PNG) [file pcbi.1005285.s003.png]
